# Sensorineural Hearing Loss Affects Functional Connectivity of the Auditory Cortex, Parahippocampal Gyrus and Inferior Prefrontal Gyrus in Tinnitus Patients

**DOI:** 10.3389/fnins.2022.816712

**Published:** 2022-04-01

**Authors:** Junming Chen, Yuanxin Zhao, Tuanming Zou, Xiaoling Wen, Xiaowei Zhou, Youjun Yu, Zhen Liu, Meige Li

**Affiliations:** Department of Otolaryngology, The First People’s Hospital of Foshan, Foshan, China

**Keywords:** tinnitus, sensorineural hearing loss, event-related potential, Granger causality, functional connectivity

## Abstract

**Background:**

Tinnitus can interfere with a patient’s speech discrimination, but whether tinnitus itself or the accompanying sensorineural hearing loss (SNHL) causes this interference is still unclear. We analyzed event-related electroencephalograms (EEGs) to observe auditory-related brain function and explore the possible effects of SNHL on auditory processing in tinnitus patients.

**Methods:**

Speech discrimination scores (SDSs) were recorded in 21 healthy control subjects, 24 tinnitus patients, 24 SNHL patients, and 27 patients with both SNHL and tinnitus. EEGs were collected under an oddball paradigm. Then, the mismatch negativity (MMN) amplitude and latency, the clustering coefficient and average path length of the whole network in the tinnitus and SNHL groups were compared with those in the control group. Additionally, we analyzed the intergroup differences in functional connectivity among the primary auditory cortex (AC), parahippocampal gyrus (PHG), and inferior frontal gyrus (IFG).

**Results:**

SNHL patients with or without tinnitus had lower SDSs than the control subjects. Compared with control subjects, tinnitus patients with or without SNHL had decreased MMN amplitudes, and SNHL patients had longer MMN latencies. Tinnitus patients without SNHL had a smaller clustering coefficient and a longer whole-brain average path length than the control subjects. SNHL patients with or without tinnitus had a smaller clustering coefficient and a longer average path length than patients with tinnitus alone. The connectivity strength from the AC to the PHG and IFG was lower on the affected side in tinnitus patients than that in control subjects; the connectivity strength from the PHG to the IFG was also lower on the affected side in tinnitus patients than that in control subjects. However, the connectivity strength from the IFG to the AC was stronger in tinnitus patients than that in the control subjects. In SNHL patients with or without tinnitus, these changes were magnified.

**Conclusion:**

Changes in auditory processing in tinnitus patients do not influence SDSs. Instead, SNHL might cause the activity of the AC, PHG and IFG to change, resulting in impaired speech recognition in tinnitus patients with SNHL.

## Introduction

Tinnitus is one of the most common clinical symptoms in otology and refers to the sensation of abnormal sound signals in the ear or brain without an external sound source or electrical stimulation (Malouff et al., 2011). Its prevalence in adults is approximately 10–15%, especially in those with sensorineural hearing loss (SNHL) (Zeng et al., 2020). Many patients with tinnitus complain about hearing difficulty, such as poor speech perception in noise, which may be due to changes in central activity caused by tinnitus itself (Moon et al., 2015) or SNHL (Zeng et al., 2020). Exploring the effects of tinnitus and SNHL on central auditory processing in tinnitus patients could be helpful in revealing the central causes of speech recognition difficulty and would be of great significance for advancing the rehabilitation treatment of tinnitus and improving quality of life.

It has been suggested that patients with tinnitus have auditory processing disorders responsible for the decreases in speech recognition ability. Yang et al. (2013) observed that in an oddball stimulus pattern, the amplitude of mismatch negativity (MMN) induced by different frequencies of pure-tone stimulation in patients with tinnitus decreased, which was due to plastic changes in the auditory centers and the weakening of attention to new stimulation in the temporal lobe according to the sensory memory mechanism. Mohebbi et al. (2019) further found that the amplitudes of tinnitus patients’ MMNs induced by notched sounds of different frequencies were decreased. They hypothesized that tinnitus interferes with the formation of auditory memory, resulting in dysfunction of central processing associated with detection of a mismatch between internal expectations and the incoming information according to the theory of predictive coding framework. In this framework, afferent inputs are communicated bottom-up via forward connections from the auditory cortices, while predictions about these inputs are communicated top-down via backward connections from higher brain areas. In tinnitus patients, the central nervous system might not compare a new stimulus with auditory memories, so the ability to distinguish differences in sounds is weakened. Studies have suggested that the brain distinguishes the differential stimulus in an oddball stimulus pattern within the MMN, which involves the auditory center, memory center and frontal lobe (Fitzgerald and Todd, 2020). Thus, investigating the characteristics of central activity during this period and exploring the mechanism underlying changes in central auditory processing would provide greater insight. When the information provided by the amplitude or latency of several electrodes is insufficient, another method, such as graph-theoretic analysis, is needed to observe the subtle changes in the signal obtained from whole-brain electroencephalography (EEG) electrodes (Mohan et al., 2018).

EEG graph-theoretic analysis has been widely used to observe the functional connectivity of the whole brain and shed light on the possible mechanisms underlying auditory processing disorders. Mohan et al. (2018) observed the functional connectivity of the brain in patients with tinnitus with resting-state EEG and found that the clustering coefficient of the whole brain was decreased in patients, indicating that the overall functional connections among different brain centers was weakened in the resting state in patients with tinnitus. Mohan et al. (2016) observed that the clustering coefficient of low-frequency EEG signals in patients with tinnitus decreased and the length of the characteristic path increased, indicating that the central activity of patients with tinnitus shifted from a small-world network to a more regular network. However, the adaptability and the ability to respond to different stimuli decreased, which was also reflected by a decline in the connections between the auditory centers and other centers. The above results were obtained in the resting state, however; to investigate the mechanisms underlying auditory processing in patients with tinnitus, event-related functional connectivity analysis is needed.

Source analysis of EEG signals can further be implemented to observe the activity of different brain regions and reveal the mechanisms underlying central auditory processing impairments in tinnitus patients. Standardized low-resolution brain electrical tomography (sLORETA) is an algorithm that can be used to display the activity of neurons in three-dimensional space. It uses EEG data to calculate the inverse solution of the EEG to obtain the parameters of neuronal electrical activity. This method has less error and higher accuracy in calculating the central source of EEG components (Vanneste and De Ridder, 2015). By using resting-state EEG and sLORETA, De Ridder and Vanneste (2014) found that the connection between the auditory cortex (AC) and parahippocampal gyrus (PHG) was enhanced in patients with tinnitus. They speculated that tinnitus signals were transmitted from the AC to the PHG but that the adaptability of the PHG to tinnitus signals was decreased, which led to continuous activation of neurons in this area and suggesting that the function of the AC and PHG in patients with tinnitus was altered. Zhang et al. (2020) applied sLORETA to resting-state EEG and found that the connectivity of the AC, PHG, and angular gyrus, associated with auditory semantic processing, was enhanced in patients with tinnitus, suggesting that the AC and PHG are involved in auditory processing in patients with tinnitus. In addition, by analyzing resting-state EEG signals through sLORETA and Granger causality analysis, Mohan et al. (2018) found that the influence of the auditory center on inferior prefrontal gyrus (IFG) function was weakened in patients with tinnitus. They presumed that the ability of the AC to transmit auditory information to the IFG was weakened. Rogers and Davis (2017), using functional magnetic resonance imaging (fMRI), found that the IFG was able to receive and differentiate information from the AC and other centers.

However, previous studies have included tinnitus subjects with or without SNHL. SNHL was thought to be involved in central auditory processing dysfunction (Zeng et al., 2020), but the effects of tinnitus and SNHL on speech discrimination remain unclear. Given the above information, we hypothesized that differences in functional connectivity among the AC, PHG, and IFG in tinnitus patients are either due to tinnitus or tinnitus-associated SNHL, which subsequently affects their speech recognition ability. In this study, MMN and EEG graph-theoretic analyses were used to observe the brain function of tinnitus patients, and sLORETA was used to analyze functional connectivity of the auditory processing-related cortex through event-related EEG in a passive listening paradigm to explore possible the mechanisms underlying auditory processing impairment in tinnitus patients.

## Materials and Methods

### Participants

The inclusion criteria were as follows: age 18–50 years; right-handedness; otoscopy showing a normal external auditory canal and tympanic membrane; and no vertigo or history of nervous system, mental or other systemic diseases. The exclusion criteria were as follows: local causes of tinnitus; acoustic neuroma requiring surgical or other treatment; the presence of serious diseases, such as hypertension, diabetes, hyperlipidemia, menopausal syndrome, patients that required medical treatment; serious psychological disorders that required professional psychological treatment; objective tinnitus and severe hearing hypersensitivity; and unilateral or bilateral hearing loss above 60 dB HL. Seventy-five patients aged 18–50 years with unilateral tinnitus or SNHL were selected. The course of tinnitus or SNHL was at least 6 months. There were 24 tinnitus patients without hearing loss, 11 patients with right tinnitus, and 13 patients with left tinnitus. There were 24 patients with SNHL without tinnitus, 12 patients with right SNHL, and 12 patients with left SNHL. There were 27 patients with tinnitus and ipsilateral SNHL, 12 patients with right tinnitus and hearing loss, and 15 patients with left tinnitus and hearing loss. Twenty-one volunteers aged 18–50 years without tinnitus or hearing loss and who were well matched to the patient group in terms of age and sex were recruited as the control group (Table 1). Pure tone audiometry in this group showed that the average hearing threshold was less than 25 dB HL in the range of 125–8,000 Hz, and acoustic immittance tests showed that middle ear function was normal.

**TABLE 1:** Sex, age, PTA, SDS, and MMN of the tinnitus patients and healthy control subjects.

		Control group	Tinnitus group	Hearing loss group	Tinnitus+hearing loss group
**Gender (Male:Female)**	Right	5:5	6:5	5:7	5:7
	Left	5:5	4:5	6:6	6:8
**Age (Year)**					
	Right	39.1 ± 9.3	36.7 ± 4.6	36.5 ± 6.6	37.1 ± 7.0
	Left	39.1 ± 9.3	39.3 ± 8.7	36.8 ± 8.0	33.3 ± 9.0
**PTA (dBHL)**					
	Right	13.1 ± 5.4	17.6 ± 4.2	44.2 ± 7.6*	39.3 ± 7.8*
	Left	15.00 ± 4.7	16.2 ± 5.8	41.3 ± 6.8*	40.4 ± 8.5*
**SDS (%)**					
	Right	100 ± 0	100 ± 0	80.8 ± 9.6*	83.3 ± 10.7*
	Left	100 ± 0	100 ± 0	84.8 ± 8.4*	88.0 ± 10.1*
**Amplitude (μV)**					
	Right	−2.3 ± 0.6	−1.1 ± 0.3*	−1.0 ± 0.2*	−1.1 ± 0.3*
	Left	−2.3 ± 0.6	−1.0 ± 0.5*	−0.8 ± 0.2*	−0.9 ± 0.3*
**Latency (ms)**					
	right	213.0 ± 8.9	215.5 ± 7.9	278.0 ± 5.8**	282.6 ± 5.1**
	left	213.0 ± 8.9	214.2 ± 7.8	280.8 ± 4.3**	276.1 ± 5.5**

*p < 0.05 vs. control group. **p < 0.05 vs. Tinnitus group.

### Speech Discrimination Score

The speech discrimination score (SDS) test was performed using a speech audiometer (AD229e, Interacoustics, Denmark), and the stimuli were presented monaurally to the test ear via an inserted earphone (TDH-39, Denmark). The SDS was measured at a presentation level of a pure-tone average of 0.5–4 kHz and +40 dB. The SDS was obtained by calculating the correct percentage on a 50-word list of phonetically balanced consonant-nucleus-consonant Mandarin words (Chen et al., 2016). The range of possible SDS values was 0–100.

### Electroencephalogram Data Recording and Preprocessing

A 256-channel auditory event-related potential (ERP) instrument (Net Amps 400, EGI, United States) was used. During the test, the subjects were asked to take a sitting position and relax. The subjects read magazines or newspapers and were asked to not pay attention to the stimulus during the test. A passive oddball paradigm was used (Chen et al., 2016), with a sampling rate of 250 Hz. Before recording, the subjects were asked to clean their scalp and wear electrode caps. The resistance of the electrodes was maintained below 40 kΩ (American Clinical Neurophysiology Society, 2006). The speech sounds/ba/and/da/were used as stimuli, and each block included 1,000 stimulations. The standard stimulus was/ba/, which was presented a total of 850 times (85%), and the deviant stimulus was/da/, which was presented a total of 150 times (15%). The interstimulus interval was 750 ms. There were 60 presentations of the standard stimulus/ba/before presentation of the first deviant stimulus/da/, and at least two standard stimuli were presented before each deviant stimulus. The stimuli were controlled by Eprime 2.0 software (Psychology software tools, Inc., United States) and delivered through a loudspeaker placed 1 meter in front of the subject’s head at a sound intensity of approximately 40 dB above the threshold of the worst ear or at a comfortable level (near both ears) under conditions without a hearing aid. Offline analysis of the recorded raw EEG data included the following: filtering (0.1∼30 Hz), EEG segmentation (analysis time: −100 to + 600 ms), artifact removal (eye movement, eye opening, bad channel), bad electrode replacement, superposition average, reference electrode selection (using the nasal root reference electrode), and baseline correction. The MMN is an auditory ERP produced in response to the presentation of a deviant stimulus after repeated exposure to standard stimuli. In this study, the MMN was identified as a negative component in the range of 150–300 ms calculated by subtracting the deviant waveform from the standard waveform (Liang et al., 2014). The peak negative values of the MMN at the Fz electrodes in a defined time window were identified by a computer algorithm.

### Brain Function Network Calculation

MATLAB r2018b software (MathWorks, United States) was used to read the signals. The calculation of the brain function network involved the following: each channel of ERP data was defined as a node of the network, and there were 257 electrodes (including the reference electrode); thus, the number of nodes (n) in the ERP network was 257. The correlation coefficient matrix of the time series (150–300 ms) (Chen et al., 2016) in the deviant waveform where the MMN was located was used to represent the relationship between the network nodes, and each element of the matrix Cij represented the correlation value between the network nodes i and j. When a correlation value was greater than the threshold value (0.95), the two nodes were considered to be functionally related, and the matrix element value of the brain functional network was 1; otherwise, for two nodes that were considered to be functionally independent, the matrix element value of the brain functional network was 0.

In this study, the synchronization likelihood (SL) method was used to calculate the synchronous likelihood value SLxy between two electrodes (x and y) as the correlation between the network nodes (C). The SL method was used for pairwise estimation of dynamic functional connectivity. SL identifies non-linear statistical interdependencies between a pair of signals and is by its nature dynamic, normalized and seemingly unaffected by non-stationarity. These properties make SL a suitable tool for functional connectivity studies using EEG measurements, as EEG signals are often considered non-stationary, and the functional coupling between different neuronal ensembles is non-linear (Racz et al., 2018). The synchronous likelihood value SL between two electrodes was calculated according to Racz et al. (2018).

SL measures the general synchronization between discretely sampled processes x(t) and y(t), t = 1, 2, … N. First, the temporal evolution of x(t) and y(t) is reconstructed in the state space by temporal embedding, where x(t) and y(t) is converted into a set of state space vectors X(t) and Y(t) as


X(t)=x(t,t-m,t-2m,……,t-(d-1)m),

Y(t)=Y(t,t-m,t-2m,……,t-(d-1)m),

where x and y represent the electrode channels; N represents the length of time and was set to 150 according to the 150–300 ms time window in our study; d (embedding dimension) = 3.6; m (time lag) = 4 ms. The probability for every state space vector X (t) and Y(t) and the distance of a randomly selected vector X(t+u) is closer than distance rx(t) as


$$\begin{array}{c} C\left(\mathrm{rx}\left(t\right),X\right)=\frac{1}{2\left(w2-w1\right)}\sum_{w1<\left|u\right|<w2}^{N}\theta \{\text{rx}\left(\text{t}\right)\\ \;-\left|X\left(t\right)-X\left(t+u\right)\right|\} \end{array}$$

where u is the temporal distance, |⋅| is the Euclidean norm, θ is the Heaviside step function, w1 is the Theiler correction for autocorrelation and w2 is a window parameter such as w1 < w2 < N. w2 serves as the time window in a sliding-window analysis, and as u can be negative as well. In our study, w1 was set to 20.8 ms; and w2 was set to 30 ms. For data with time and frequency information, we used a filtering range of 0.1–30 Hz for data processing according to Montez et al. (2006).

The distance parameters rx(t) and ry(t) are set for every time point t that C [rx (t), X] = C[ry(t),Y] = pref, where pref = 0.05. Finally, the synchronization likelihood at time point t is defined as the conditional probability that Y(t) and Y(t+u) are closer than ry(t) given that X(t) and X(t+u) are closer than rx(t) and calculates as


$$\begin{array}{c} \mathrm{SL}\left(t\right)=\frac{1}{2pref\left(\mathrm{w2}-\mathrm{w1}\right)}\times \sum_{\mathrm{w1}<\left|\text{u}\right|<\mathrm{w2}}^{\text{N}}\theta \{\mathrm{rx}\left(t\right)-|\text{X}\left(\text{t}\right)\\ \;-\text{X}\left(\text{t}+\text{u}\right)|)\times \theta \left(\mathrm{ry}\left(t\right)-|\text{Y}\left(\text{t}\right)-\text{Y}\left(\text{t}+\text{u}\right)|\right\} \end{array}$$

The synchronous likelihood value SL between two electrodes was used to calculate the clustering coefficient, an important statistical feature of complex networks. The clustering coefficient is calculated by estimating the fraction of the number of triangles formed around a node with its two nearest neighbors, with a larger value indicating greater functional connectivity between nodes. Assuming that node i is connected with k(i) other nodes, there may be at most k(i)(k(i) - 1)/2 edges between these k(i) nodes; given E(i) actual edges between the nodes, the clustering coefficient of node i is calculated as follows (Hong et al., 2016):


$$C\left(i\right)=\frac{2E\left(i\right)}{k\left(i\right)\left(k\left(i\right)-1\right)}.$$

The clustering coefficient (C) of the whole network is calculated as follows (Hong et al., 2016):


$$C=\frac{\sum_{i=1}^{n}C\left(i\right)}{n}.$$

In a complex network, different nodes can have different ways of connecting by passing through different edges. These edges are called paths, and the number of edges is called the path length. The path length estimates the ability of the network to rapidly combine information from distinct and distant brain areas, with shorter paths indicating a greater ability. From node i to node j, the number of edges that need to be passed is the length of the path Lij, and the average path length of the whole network is expressed as follows (Boersma et al., 2011):


$$Lw=\frac{1}{\left(1/N\left(N-1\right)\right)}\sum_{i=1}^{N}\sum_{j\neq 1}^{N}1/Lij.$$

### Functional Connectivity Analysis

Geosource 3.0 software of the Net Station 4.3 analysis system (EGI, United States) was used. The finite difference model (FDM) was used for sLORETA analysis. Initially, 2,447 dipoles were distributed across the cerebral cortex. After sLORETA analysis, the dipole current density was read by MATLAB r2018b software (MathWorks, United States), and the GCCA toolbox (Seth, 2010) was imported into the software for Granger causality analysis of the effective connections between the 84 Brodmann areas (BAs). Granger causality reflects the strength of effective connectivity from one region to another by quantifying how much the signal in the seed region can predict the signal in the target region. Functional connections among the AC (BA 41), PHG (BA 36), and IFG (BA 46) were observed. By calculating the Granger causality of the dipole current density of brain regions in the MMN time series (150–300 ms), we can understand the functional connectivity of brain regions in the time series (Schoffelen and Gross, 2009). The main process was as follows:

Xt and Yt represent the time series of the dipole current density of different brain regions, and their linear autoregression models are as follows:


$${\text{X}}_{\text{t}}=\sum_{\text{j}=1}^{\infty }{\text{a}}_{1j}{\text{X}}_{\text{t-j}}+\varepsilon_{1t}var\left(\varepsilon_{1t}\right)=\sum_{1},$$

$${\text{Y}}_{\text{t}}=\sum_{\text{j}=1}^{\infty }{\text{d}}_{1j}{\text{Y}}_{\text{t-j}}+\eta_{1t}var\left(\eta_{1t}\right)=\Gamma_{1},$$

where a_1*j*_ and d_1*j*_ are the coefficients of the autoregression model, ε_1*t*_ and η_1*t*_ represent the noise term, and Σ_1_ and Γ_1_ represent the variances in the noise term. The size of the noise term depends only on the past time values of Xt and Yt. The joint regression models of Xt and Yt can be expressed as follows:


$${\text{X}}_{\text{t}}=\sum_{\text{j}=1}^{\infty }{\text{a}}_{2j}{\text{X}}_{\text{t-j}}+\sum_{\text{j}=1}^{\infty }{\text{b}}_{2j}{\text{Y}}_{\text{t-j}}+\varepsilon_{2t}var\left(\varepsilon_{2t}\right)=\sum_{2},$$

$${\text{Y}}_{\text{t}}=\sum_{\text{j}=1}^{\infty }{\text{c}}_{2j}{\text{Y}}_{\text{t-j}}+\sum_{\text{j}=1}^{\infty }{\text{d}}_{2j}{\text{Y}}_{\text{t-j}}+\eta_{2t}var\left(\eta_{2t}\right)=\Gamma_{2}.$$

Among these variables, a_2*j*_, b_2*j*_, c_2*j*_ and d_2*j*_ represent the coefficients of the joint regression model, and ε_2*t*_ and η_2*t*_ are error terms, which are independent of each other in time. The definition of the Granger causality of Yt by Xt can be expressed as follows:


$${\text{F}}_{\text{Y}\to \text{X}}=\ln\frac{\sum_{1}}{\sum_{2}}.$$

When F_*Y*→*X*_ > 0, brain region Y has a functional influence on brain region X; when F_*Y*→*X*_ ≤ 0, brain region Y has no functional influence on brain region X. Similarly, the Granger causality of F_*X*→*Y*_ is defined as follows:


$${\text{F}}_{\text{X}\to \text{Y}}=\ln{\frac{\Gamma_{1}}{\Gamma }}_{2}.$$

When F_*X*→*Y*_ > 0, brain region X has a functional effect on brain region Y; when F_*X*→*Y*_ ≤ 0, brain region X has no functional effect on brain region Y.

### Statistical Analysis

The SDS, MMN amplitude and latency, clustering coefficient, average path length and Granger causality among different groups were compared by one-way ANOVA. The false discovery rate (FDR) was used to correct the *p*-value after comparing the Granger causality values for different brain regions. *p* < 0.05 was considered statistically significant.

## Results

We found that there was no difference in the SDS of tinnitus patients without SNHL and those of control subjects (Table 1), but the SDS in patients of SNHL with or without tinnitus was lower than that of control subjects (Table 1).

**FIGURE 1:**
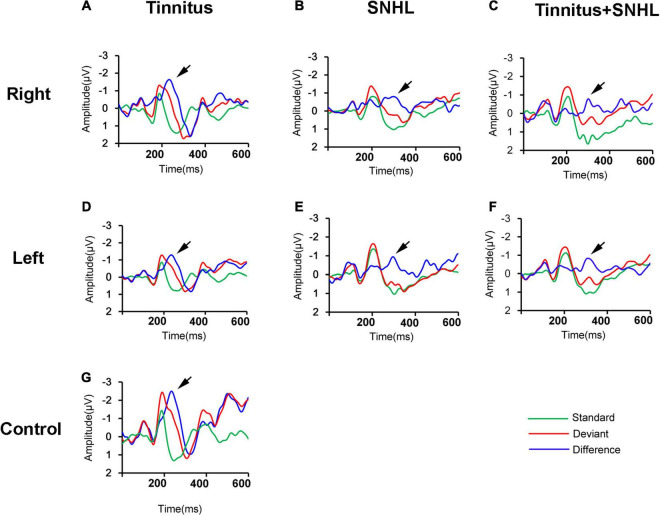
**(A–F)** The grand averaged waveforms of patients with tinnitus, SNHL and tinnitus/SNHL of the right and left ears, respectively. **(G)** The grand averaged waveforms of the control subjects. All waveforms were recorded from the Fz electrode. Standard: response to standard stimuli; Deviant: response to deviant stimuli; Difference: difference waveforms. The black arrow indicates the MMN.

To explore possible mechanisms underlying the decrease in the SDS in SNHL patients with or without tinnitus, we first observed the changes in the MMN in tinnitus patients with or without SNHL. We found that compared to those of control subjects (Figure 1G), the MMN amplitude (Table 1 and Figures 1A,D) at the Fz electrodes decreased, but latency (Table 1 and Figures 1A,D) remained unchanged in tinnitus patients without SNHL. Compared to those of control subjects, the MMN amplitude (Table 1) decreased and latency (Table 1) increased in SNHL patients with (Figures 1C,F) or without tinnitus (Figures 1B,E).

**FIGURE 2:**
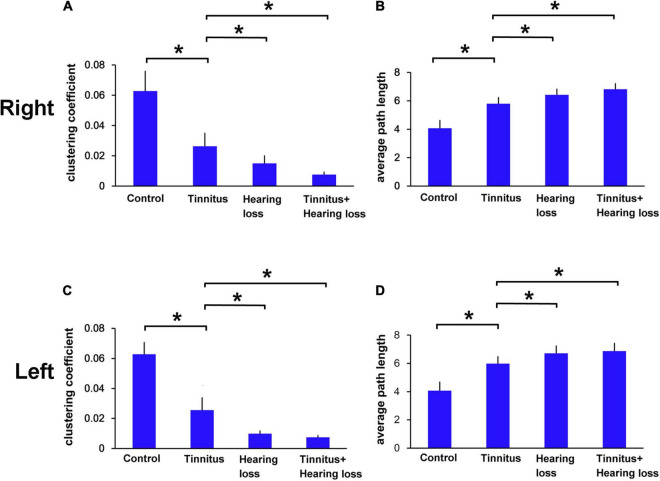
**(A–D)** Comparison of the clustering coefficients and average path lengths for the right and left ears among the control, tinnitus and tinnitus/SNHL groups. *p < 0.05.

We then observed the functional connectivity at EEG level in patients with tinnitus and SNHL during the MMN time series of deviant sound processing. We found that tinnitus patients with or without SNHL had a smaller clustering coefficient value (Figures 2A,C) and a longer average path length (Figures 2B,D) than control subjects. SNHL patients with or without tinnitus had smaller clustering coefficients (Figures 2A,C) and a greater average path length than tinnitus patients without SNHL (Figures 2B,D).

**FIGURE 3:**
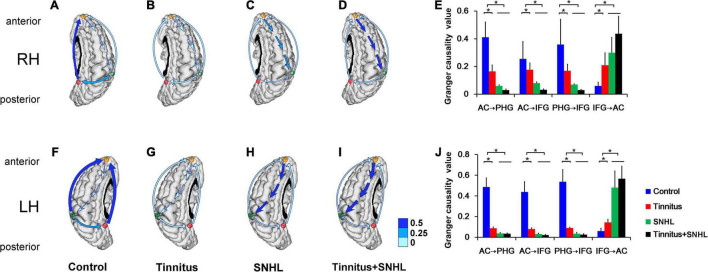
**(A–D)** The right brain of the control group, right-ear tinnitus group, right-ear SNHL group and right-ear tinnitus/SNHL group, respectively. **(E)** Comparison of the functional connectivity of the AC, PHG, and IFG between right-ear tinnitus, SNHL and control subjects. **(F–I)** The left brain of the control group, left-ear tinnitus group, left-ear SNHL group and left-ear tinnitus/SNHL group, respectively. **(J)** Comparison of the functional connectivity of the AC, PHG, and IFG between left-ear tinnitus, SNHL and control subjects. The green, red and orange dots represent the AC, PHG, and IFG, respectively. The blue arrow represents connectivity. RH, Right cerebral hemisphere; LH, Left cerebral hemisphere. *P < 0.05.

To investigate how the function of the brain region of interest changed in the MMN time series of auditory deviant processing, we then observed the relationship between the AC, PHG, and IFG in tinnitus and SNHL patients with sLORETA combined with Granger causality analysis. The results showed that connectivity strength from the AC to the PHG and IFG on the tinnitus side in tinnitus patients without SNHL (Figures 3B,G,E,J) was lower than that in control subjects (Figures 3A,F); the connectivity strength from the PHG to the IFG on the tinnitus side was lower in tinnitus patients without SNHL (Figures 3B,G,E,J) than in control subjects (Figures 3A,F); and the connectivity strength from the IFG to the AC on the tinnitus side in tinnitus patients without SNHL (Figures 3B,G,E,J) was stronger than that in control subjects (Figures 3A,F). In SNHL patients with (Figures 3D,I,E,J) or without (Figures 3C,H,E,J) tinnitus, the connectivity strength from the AC to the PHG and IFG on the hearing loss side was weaker than that in tinnitus patients without SNHL (Figures 3B,G), the connectivity strength from the PHG to the IFG on the hearing loss side (Figures 3C,D,H,I) was weaker than that in the tinnitus group without hearing loss (Figures 3B,G), and the connectivity strength from the IFG to the AC on the hearing loss side (Figures 3C,D,H,I) was stronger than that in the tinnitus group without hearing loss (Figures 3B,G).

We did not find differences in the connectivity strength from the AC, PHG, and IFG on the tinnitus or SNHL side to the AC, PHG, and IFG on the contralateral side between the tinnitus or SNHL subjects and the controls after multiple comparisons correction by using FDR (data not shown).

## Discussion

### Sensorineural Hearing Loss Is Associated With Declines in the Speech Discrimination Scores

We found that the SDS of tinnitus patients without hearing loss was not different from that of control patients, which is consistent with the observation of Zeng et al. (2020). Zeng et al. (2020) found that hearing loss but not tinnitus is associated with frequency discrimination and concluded that tinnitus does not interfere with speech perception in general; rather, hearing loss impairs suprathreshold processing, which directly contributes to hearing difficulty. Therefore, the SDS of tinnitus patients without SNHL obtained in a quiet context did not differ from those of control patients.

However, we found that the SDS of SNHL patients with or without tinnitus was lower than that of the control group patients. Kurioka et al. (2020) reported that even with unilateral SNHL, the maximum speech recognition score of the affected ear was lower than that of the control ear. Chen et al. (2018) observed the central activity characteristics of patients with SNHL by resting-state fMRI and found that the activities of the superior temporal gyrus, PHG, precuneus and inferior parietal lobule were decreased, while those of the middle frontal gyrus, cuneiform gyrus, and posterior central gyrus were increased in patients, suggesting that SNHL leads to changes in central activity in multiple auditory processing regions. Therefore, we propose that changes in auditory-related central activities result in a decline in speech processing ability in patients with SNHL.

### Tinnitus and Sensorineural Hearing Loss Are Associated With Changes in Mismatch Negativity

We found that the amplitude of the MMN decreased in patients with tinnitus without SNHL. Mohebbi et al. (2019) compared standard to deviant stimuli; however, possibly due to difficulty accessing memory during the comparisons, the response to the frequency and the silent gap deviant stimulus decreased as the MMN amplitude decreased in tinnitus patients. We further identified a decreased MMN amplitude and an increased MMN latency in SNHL patients with or without tinnitus. MMN amplitude has been suggested to be a neurophysiological marker of cortical auditory discrimination capacity in SNHL patients (Liang et al., 2014). In our previous study, we also discovered a longer MMN latency in patients with SNHL, which suggests that the changes in central auditory processing decreased speech discrimination ability (Chen et al., 2016). We found that SNHL might have larger impact on central auditory discrimination of variant sounds than tinnitus. We further used EEG graph-theoretic analysis to observe the specific effects of these diseases on central auditory processing.

### Larger Changes in Sensor Level Functional Connectivity in Sensorineural Hearing Loss Patients

We found that the whole-brain clustering coefficients of tinnitus patients decreased during the processing of deviant stimuli. Mohan et al. (2016) observed that the whole-brain clustering coefficient in tinnitus patients is significantly lower than that of the control at the delta, theta, and alpha frequency bands and inferred that the brain of tinnitus patients became more efficiently organized to focus only on the tinnitus sound and lost flexibility or adaptiveness, resulting in a decrease in the ability of the cerebral cortex to respond to sound stimulation. We also found that in SNHL patients with or without tinnitus, the whole-brain clustering coefficient was further decreased. Bidelman et al. (2019) found that in patients with SNHL, the whole-brain clustering coefficient decreased through event-related EEG. They reasoned that even mild degrees of SNHL produced broad neural reorganization at the full-brain level, and SNHL patients had less efficient or integrated information exchange than subjects with normal hearing. Therefore, our results indicate that compared with tinnitus, SNHL might greatly reduce the sensor level functional connectivity during the processing of deviant sounds.

We found that the whole-brain average path length in tinnitus patients was lengthened in the MMN time series when processing different stimuli. Han et al. (2018) found that the number of active central areas of tinnitus patients without SNHL were increased while the efficiency decreased, suggesting that multiple centers participated in information processing. Therefore, we presumed that for tinnitus patients, information transmission could be achieved only through more brain activity, and effective transmission of deviant information to other centers is difficult, resulting in an increase in the average path length in an event-related state. Additionally, we found that in patients with SNHL, the average path length further increased. Bidelman et al. (2019) discovered that the brain of SNHL patients might have to exert greater effort to achieve the same level of performance, thereby requiring more brain regions for auditory processing due to the compensatory central activity, but the information transmission ability was weakened. Hence, our results indicate that compared with tinnitus, SNHL might greatly reduce the information transmission ability of the whole brain during the processing of deviant sounds.

### Functional Connectivity Change in the Auditory Cortex, Parahippocampal Gyrus, and Inferior Frontal Gyrus in Tinnitus Patients

We found that the influence of the AC on the PHG and IFG and the influence of the PHG on the IFG in tinnitus patients was weakened according to event-related EEG. Hong et al. (2016) found that the connection between the AC and PHG was reduced in patients with tinnitus without hearing loss, seemingly due to increased AC processing of tinnitus intensity. This enhancement hindered the auditory center from transmitting deviant sound information to the memory center; thus, the influence of the auditory center on the memory center was weakened when processing different stimuli. Ahn et al. (2017) inferred that the decrease or absence of functional connectivity from the AC to the IFG in the tinnitus group without SNHL likely reflected a deficiency in integrated action during cognitive processing. Hong et al. (2016) also considered that it was likely that patients with tinnitus have cognitive deficits in auditory memory processing, which might result in the decrease of functional connectivity from the PHG to the prefrontal cortex in a passive listening paradigm. Thus, tinnitus patients without SNHL might have altered functional connectivity among the AC, PHG, and IFG.

Nevertheless, we found that the effect of the IFG on the AC was enhanced in tinnitus patients. The frontal lobe is responsible for suppressing the interference of irrelevant signals. Tinnitus activates the noise reduction function of the IFG, enhancing the effect of the IFG on the AC (Chen et al., 2017). Taken together, our findings may suggest that the effect of the IFG on the auditory center in tinnitus patients was enhanced.

### Altered Functional Connectivity Among the Auditory Cortex, Parahippocampal Gyrus, and Inferior Frontal Gyrus in Sensorineural Hearing Loss Patients

We found that the functional connectivity of the AC to the PHG, AC to IFG and PHG to IFG was further weakened and the functional connectivity of the IFG to the AC was enhanced in SNHL patients compared to in those with tinnitus, which was consistent with the SDS decline.

The AC is responsible for encoding the frequency, intensity and temporal information of sound. The AC transmits information to the memory center to form auditory memory for auditory comparison and discrimination (Salvari et al., 2019). Vanneste and De Ridder (2016) examined patients with SNHL and found that due to the decrease in information input, the plasticity of the AC was altered, and its activity was much weaker than that of tinnitus patients. Therefore, our results suggest that the decrease in sound-related information transmitted from the AC to the PHG might reduce the formation of auditory memory and ability to compare sound differences, hindering auditory processing in SNHL patients. The AC also transmits information to the IFG for advanced processing, such as attention switching toward the deviant stimulus. Shang et al. (2020) conducted an fMRI and magnetoencephalographic imaging study and observed decreased spontaneous activity in patients with SNHL that resulted in a decrease in the transmission of information from the AC to the IFG. Thus, the attention to deviant stimuli may have been weakened by the AC changes in SNHL patients. Third, in the deviant stimuli processing pathways, PHG is responsible for sensory memory comparison. Decreased cerebral gray matter volume of the PHG has been associated with impaired episodic memory in SNHL patients (Yang et al., 2014). In an fMRI study, Chen et al. (2018) observed a decrease in spontaneous activity in the PHG and a decrease in connectivity from the PHG to other centers involved in complex auditory processing in SNHL patients. They inferred that these changes are associated with speech processing dysfunction.

Moreover, in patients with SNHL, we observed that the effect of the IFG on the AC was enhanced. Pereira-Jorge et al. (2018) found that after hearing loss, the plasticity of the IFG was altered, and its activity was enhanced, which reflected the enhancement of the IFG in controlling the activities of speech-related centers, including the AC. Therefore, we presumed that the inhibitory effect of the IFG on the AC was increased in patients with SNHL, which further interfered with the processing of differential acoustic signals in the AC.

Overall, we may conclude that the plasticity changes in the AC, PHG, and IFG caused by SNHL may contribute to SDS decline.

### Limitations

This study has some limitations. First, the Granger causality can be affected by the trial-to-trial variability of cortical evoked responses (Wang et al., 2008). Although this influence could be reduced through data preprocessing via the GCCA toolbox (Barnett and Seth, 2014), future research is needed to find out a better causality measures. Second, our connectivity matrices were derived from source signals, and the effects of field spread cannot be fully abolished in EEG, even at the source level. Correlated activity of adjacent sources reduces the accuracy of functional connectivity analyses. However, proper interpretation of source connectivity results can be achieved by analyzing the relative changes in connectivity caused by experimental manipulations (Bidelman et al., 2019). Because field spread effects are identical across our experimental conditions (i.e., groups and noise levels) they are canceled out and are unlikely to account for group differences. Moreover, when combined with dense array EEG with many more electrodes (up to 256), sLORETA has less error and higher accuracy for calculating the central source of EEG components (Holmes, 2008). Third, because the number of patients in our study was small, we did not compare the difference between unilateral and bilateral SNHL. Although we found differences in central functional connectivity between patients with unilateral SNHL and control patients, comparing the differences between unilateral and bilateral SNHL could further reveal the effects of tinnitus on different cerebral hemispheres. Additionally, the speech recognition ability of tinnitus subjects with or without SNHL decreases in environments with noise (Zeng et al., 2020). The lack of a difference in our study between the subjects with tinnitus without SNHL and controls likely reflects a ceiling effect through testing in quiet. The functional connectivity of the brain may be altered in other ways, which merit further study after these relationships have been established in quiet condition.

## Conclusion

In tinnitus patients, even when the SDS does not change, EEG activity related to auditory processing does. Changes in auditory processing in tinnitus patients do not influence the SDS. Instead, SNHL might cause the plasticity change in the AC, PHG, and IFG and result in speech recognition dysfunction in tinnitus patients with SNHL.

## Data Availability Statement

The original contributions presented in the study are included in the article/Supplementary Material, further inquiries can be directed to the corresponding author/s.

## Ethics Statement

The studies involving human participants were reviewed and approved by the Institutional Review Board of the First People’s Hospital of Foshan, Foshan, China. The patients/participants provided their written informed consent to participate in this study.

## Author Contributions

JC contributed to the study conception, design, data analysis and interpretation, statistical analyses, and manuscript writing. YZ contributed to the conception, design, data acquisition and interpretation, and manuscript writing. TZ and XW contributed to the data acquisition and interpretation, and manuscript writing. XZ, YY, ZL, and ML contributed to the data acquisition and interpretation, and manuscript writing. All authors read and approved the final manuscript.

## Conflict of Interest

The authors declare that the research was conducted in the absence of any commercial or financial relationships that could be construed as a potential conflict of interest.

## Publisher’s Note

All claims expressed in this article are solely those of the authors and do not necessarily represent those of their affiliated organizations, or those of the publisher, the editors and the reviewers. Any product that may be evaluated in this article, or claim that may be made by its manufacturer, is not guaranteed or endorsed by the publisher.
