# Supplementary material for: Sensorineural Hearing Loss Affects Functional Connectivity of the Auditory Cortex, Parahippocampal Gyrus and Inferior Prefrontal Gyrus in Tinnitus Patients
Source: Front Neurosci. 2022 Apr 1;16:816712. doi: 10.3389/fnins.2022.816712 (PMC9011051; doi:10.3389/fnins.2022.816712)
Supplement: Supplementary file 1 [file Table_1.DOCX]

**Data of manuscript**

**Table1** Sex, age, pure tone audiometry and speech discrimination score in patients with tinnitus or sensorineural hearing loss of the right ear.

| Group  （1=control，2=tinnitus，3=hearing loss，4=tinnitus+hearing loss） | Sex（F=female，M=male） | Age(years) | Pure tone audiometry(dBHL) | Speech discrimination score |
| --- | --- | --- | --- | --- |
| 1 | M | 32 | 2 | 100 |
| 1 | M | 50 | 20 | 100 |
| 1 | M | 47 | 20 | 100 |
| 1 | M | 42 | 19 | 100 |
| 1 | M | 49 | 10 | 100 |
| 1 | M | 38 | 15 | 100 |
| 1 | M | 18 | 23 | 100 |
| 1 | M | 31 | 18 | 100 |
| 1 | M | 36 | 13 | 100 |
| 1 | M | 48 | 10 | 100 |
| 1 | F | 36 | 9 | 100 |
| 1 | F | 43 | 15 | 100 |
| 1 | F | 45 | 14 | 100 |
| 1 | F | 44 | 10 | 100 |
| 1 | F | 36 | 10 | 100 |
| 1 | F | 23 | 3 | 100 |
| 1 | F | 47 | 10 | 100 |
| 1 | F | 47 | 18 | 100 |
| 1 | F | 40 | 10 | 100 |
| 1 | F | 48 | 11 | 100 |
| 1 | F | 23 | 16 | 100 |
| 2 | M | 39 | 10 | 100 |
| 2 | M | 40 | 23 | 100 |
| 2 | M | 40 | 22 | 100 |
| 2 | M | 31 | 20 | 100 |
| 2 | M | 42 | 12 | 100 |
| 2 | M | 34 | 18 | 100 |
| 2 | F | 42 | 18 | 100 |
| 2 | F | 36 | 16 | 100 |
| 2 | F | 28 | 20 | 100 |
| 2 | F | 33 | 21 | 100 |
| 2 | F | 39 | 14 | 100 |
| 3 | M | 44 | 40 | 70 |
| 3 | M | 35 | 48 | 80 |
| 3 | M | 35 | 60 | 70 |
| 3 | M | 34 | 41 | 80 |
| 3 | M | 37 | 35 | 100 |
| 3 | F | 29 | 48 | 80 |
| 3 | F | 34 | 34 | 100 |
| 3 | F | 34 | 42 | 80 |
| 3 | F | 35 | 46 | 80 |
| 3 | F | 46 | 50 | 70 |
| 3 | F | 49 | 36 | 80 |
| 3 | F | 26 | 51 | 80 |
| 4 | M | 40 | 36 | 70 |
| 4 | M | 35 | 42 | 80 |
| 4 | M | 49 | 32 | 90 |
| 4 | M | 45 | 53 | 80 |
| 4 | M | 34 | 40 | 80 |
| 4 | F | 44 | 40 | 70 |
| 4 | F | 41 | 42 | 90 |
| 4 | F | 34 | 55 | 70 |
| 4 | F | 39 | 33 | 80 |
| 4 | F | 28 | 35 | 100 |
| 4 | F | 31 | 32 | 100 |
| 4 | F | 26 | 32 | 90 |

**Table 2** The MMN amplitude and latency in patients with tinnitus or sensorineural hearing loss of the right ear.

| Group  （1=control，2=tinnitus，3=hearing loss，4=tinnitus+hearing loss） | Amplitude(µV) | Latency(ms) |
| --- | --- | --- |
| 1 | -1.5 | 222.8 |
| 1 | -2.2 | 225.7 |
| 1 | -1.9 | 206.8 |
| 1 | -2.1 | 201.1 |
| 1 | -2.2 | 205.7 |
| 1 | -2.2 | 213.8 |
| 1 | -2.0 | 221.4 |
| 1 | -2.5 | 196.8 |
| 1 | -2.8 | 205.6 |
| 1 | -3.1 | 228.1 |
| 1 | -2.4 | 213.4 |
| 1 | -3.1 | 210.0 |
| 1 | -1.5 | 215.7 |
| 1 | -3.6 | 219.3 |
| 1 | -1.9 | 209.7 |
| 1 | -2.1 | 223.4 |
| 1 | -1.2 | 215.6 |
| 1 | -3.1 | 224.8 |
| 1 | -2.9 | 206.2 |
| 1 | -2.2 | 206.1 |
| 1 | -3.1 | 203.1 |
| 2 | -.8 | 204.8 |
| 2 | -1.6 | 223.0 |
| 2 | -1.1 | 225.0 |
| 2 | -1.3 | 214.3 |
| 2 | -.8 | 221.3 |
| 2 | -1.6 | 217.5 |
| 2 | -1.6 | 220.9 |
| 2 | -1.0 | 222.9 |
| 2 | -.6 | 205.0 |
| 2 | -1.1 | 212.1 |
| 2 | -1.2 | 204.6 |
| 3 | -1.4 | 270.4 |
| 3 | -1.1 | 286.2 |
| 3 | -1.3 | 274.1 |
| 3 | -.8 | 280.5 |
| 3 | -1.3 | 282.3 |
| 3 | -1.1 | 280.3 |
| 3 | -.8 | 270.0 |
| 3 | -1.0 | 283.8 |
| 3 | -1.0 | 278.9 |
| 3 | -1.5 | 276.8 |
| 3 | -.6 | 283.6 |
| 3 | -.8 | 269.4 |
| 4 | -1.9 | 280.7 |
| 4 | -1.3 | 277.2 |
| 4 | -.6 | 276.3 |
| 4 | -1.0 | 290.7 |
| 4 | -.9 | 290.2 |
| 4 | -.7 | 288.9 |
| 4 | -1.3 | 280.9 |
| 4 | -.7 | 285.1 |
| 4 | -1.2 | 280.1 |
| 4 | -1.3 | 276.3 |
| 4 | -.7 | 281.5 |
| 4 | -.7 | 284.0 |

**Table3** The clustering coefficient, average path length and functional connectivity of AC, PHG and IFG in the right cerebral hemisphere in patients with tinnitus or sensorineural hearing loss of the right ear.

| Group  （1=control，2=tinnitus，3=hearing loss，4=tinnitus+hearing loss） | clustering coefficient | average path length | AC→PHG | AC→IFG | PHG→IFG | IFG→AC |
| --- | --- | --- | --- | --- | --- | --- |
| 1 | .0530 | 4.7294 | .4196 | .1556 | .2186 | .0617 |
| 1 | .0504 | 3.3348 | .3466 | .1014 | .2456 | .0421 |
| 1 | .0713 | 4.8687 | .4710 | .1900 | .5339 | .0820 |
| 1 | .0566 | 4.0226 | .5280 | .1532 | .4509 | .0855 |
| 1 | .0532 | 3.7283 | .4708 | .4192 | .4969 | .0855 |
| 1 | .0451 | 3.7807 | .5478 | .1876 | .5228 | .0717 |
| 1 | .0623 | 3.9456 | .5302 | .2154 | .2740 | .0480 |
| 1 | .0803 | 3.8453 | .2446 | .3330 | .6507 | .0062 |
| 1 | .0750 | 4.8775 | .3795 | .4130 | .2580 | .0899 |
| 1 | .0850 | 4.2549 | .5350 | .2057 | .5132 | .0582 |
| 1 | .0590 | 3.4236 | .5280 | .1579 | .1886 | .0552 |
| 1 | .0530 | 3.6165 | .2240 | .1024 | .5230 | .0439 |
| 1 | .0570 | 4.8392 | .4564 | .1779 | .1046 | .0894 |
| 1 | .0643 | 4.8232 | .4122 | .3673 | .1232 | .0915 |
| 1 | .0639 | 3.3548 | .3581 | .2189 | .4479 | .0260 |
| 1 | .0619 | 3.6618 | .4992 | .4832 | .3057 | .0727 |
| 1 | .0680 | 4.0916 | .3391 | .4995 | .1134 | .0479 |
| 1 | .0400 | 3.5000 | .2601 | .1802 | .1926 | .0808 |
| 1 | .0594 | 3.0787 | .2242 | .3474 | .1815 | .0332 |
| 1 | .0637 | 3.8802 | .5175 | .3278 | .6241 | .0903 |
| 1 | .0560 | 4.4798 | .3436 | .1179 | .5715 | .0065 |
| 2 | .0117 | 6.3763 | .1811 | .1982 | .1883 | .1906 |
| 2 | .0238 | 5.8215 | .1706 | .1919 | .1825 | .1685 |
| 2 | .0348 | 5.5123 | .1834 | .1825 | .1851 | .1782 |
| 2 | .0321 | 5.5808 | .1720 | .1931 | .1848 | .1163 |
| 2 | .0235 | 5.8100 | .1836 | .1993 | .1982 | .2650 |
| 2 | .0204 | 5.2470 | .1790 | .1948 | .1838 | .4252 |
| 2 | .0314 | 6.2143 | .1850 | .1920 | .1764 | .1753 |
| 2 | .0379 | 5.4868 | .1746 | .1880 | .1991 | .1675 |
| 2 | .0130 | 5.5964 | .1760 | .1941 | .1722 | .1130 |
| 2 | .0320 | 6.6506 | .1808 | .1868 | .1642 | .2287 |
| 2 | .0290 | 5.5308 | .0254 | .0326 | .0305 | .2801 |
| 3 | .0083 | 6.7139 | .0593 | .0769 | .0670 | .3220 |
| 3 | .0069 | 6.2194 | .0594 | .0791 | .0713 | .3795 |
| 3 | .0088 | 6.6661 | .0760 | .0745 | .0693 | .4867 |
| 3 | .0097 | 6.4089 | .0590 | .0717 | .0753 | .4459 |
| 3 | .0093 | 6.1172 | .0492 | .0749 | .0667 | .5006 |
| 3 | .0101 | 5.6085 | .0670 | .0749 | .0576 | .2367 |
| 3 | .0092 | 6.0901 | .0612 | .0810 | .0745 | .3957 |
| 3 | .0077 | 6.9851 | .0583 | .0787 | .0638 | .5036 |
| 3 | .0105 | 7.4323 | .0588 | .0632 | .0657 | .2483 |
| 3 | .0101 | 6.4256 | .0567 | .0802 | .0712 | .3795 |
| 3 | .0098 | 6.4694 | .0607 | .0684 | .0647 | .6832 |
| 3 | .0088 | 6.6654 | .0586 | .0939 | .0669 | .3465 |
| 4 | .0065 | 6.8396 | .0252 | .0379 | .0310 | .3993 |
| 4 | .0042 | 7.0290 | .0336 | .0237 | .0283 | .4644 |
| 4 | .0095 | 6.3329 | .0322 | .0351 | .0249 | .6405 |
| 4 | .0064 | 6.2807 | .0440 | .0383 | .0375 | .3550 |
| 4 | .0097 | 6.9692 | .0290 | .0291 | .0291 | .5111 |
| 4 | .0097 | 7.4411 | .0274 | .0310 | .0367 | .3331 |
| 4 | .0072 | 6.7417 | .0208 | .0299 | .0266 | .3878 |
| 4 | .0076 | 7.4524 | .0315 | .0208 | .0223 | .7066 |
| 4 | .0063 | 6.8323 | .0340 | .0266 | .0330 | .4417 |
| 4 | .0091 | 6.9408 | .0370 | .0397 | .0350 | .2910 |
| 4 | .0082 | 6.6638 | .0320 | .0354 | .0296 | .3556 |
| 4 | .0078 | 6.3234 | .0132 | .0354 | .0296 | .3556 |

**Table4** The functional connectivity from the right hemisphere to the left hemisphere in patients with tinnitus or sensorineural hearing loss of the right ear.

| Group  （1=control，2=tinnitus，3=hearing loss，4=tinnitus+hearing loss） | AC→PHG | AC→IFG | PHG→IFG | IFG→AC |
| --- | --- | --- | --- | --- |
| 1 | .5450 | .1646 | .2340 | .0287 |
| 1 | .6264 | .1862 | .2500 | .0864 |
| 1 | .4841 | .0689 | .2984 | .0677 |
| 1 | .2657 | .4355 | .4041 | .0541 |
| 1 | .4894 | .2682 | .3001 | .0895 |
| 1 | .4099 | .3285 | .2743 | .0682 |
| 1 | .5448 | .3556 | .4701 | .0615 |
| 1 | .3867 | .1035 | .2471 | .0495 |
| 1 | .5420 | .1543 | .3455 | .0938 |
| 1 | .3772 | .2087 | .4321 | .0210 |
| 1 | .4446 | .2307 | .5536 | .0547 |
| 1 | .4706 | .2383 | .2568 | .0440 |
| 1 | .3001 | .2886 | .1751 | .0691 |
| 1 | .4783 | .2205 | .3703 | .0849 |
| 1 | .2231 | .3360 | .3921 | .0607 |
| 1 | .4092 | .1722 | .2514 | .0433 |
| 1 | .4225 | .1871 | .3445 | .0509 |
| 1 | .3701 | .3211 | .3807 | .0549 |
| 1 | .4818 | .3263 | .1750 | .0721 |
| 1 | .3464 | .3018 | .3190 | .0682 |
| 1 | .3565 | .1316 | .3586 | .0895 |
| 2 | .3140 | .1185 | .3933 | .0504 |
| 2 | .4205 | .2523 | .5346 | .0537 |
| 2 | .6807 | .2435 | .3067 | .0677 |
| 2 | .1259 | .3500 | -.0002 | .0885 |
| 2 | .3884 | .1923 | .2073 | .0521 |
| 2 | .2850 | .4454 | .5510 | .0809 |
| 2 | .2162 | .0482 | .3207 | .0570 |
| 2 | .4660 | .3203 | .2883 | .0717 |
| 2 | .5860 | .2093 | .3088 | .0382 |
| 2 | .6291 | .4986 | .3257 | .0705 |
| 2 | .4014 | .3997 | .3808 | .0522 |
| 3 | .3152 | .3590 | .6993 | .1077 |
| 3 | .5769 | .2250 | .3547 | .0495 |
| 3 | .2772 | .3933 | .4655 | .0591 |
| 3 | .4405 | .3323 | .5043 | .0337 |
| 3 | .3814 | .1831 | .2303 | .0576 |
| 3 | .3842 | .2325 | .2090 | .0532 |
| 3 | .5466 | .3457 | .3694 | .0758 |
| 3 | .2692 | .3666 | .3482 | .0498 |
| 3 | .5608 | .2882 | .1135 | .0756 |
| 3 | .4425 | .2165 | .3128 | .0755 |
| 3 | .2977 | .2552 | .3364 | .0573 |
| 3 | .3784 | .3256 | .3895 | .0652 |
| 4 | .4055 | .0630 | .3089 | .0405 |
| 4 | .6448 | .3042 | .2243 | .1046 |
| 4 | .6090 | .1485 | .2965 | .0349 |
| 4 | .4889 | .2446 | .1777 | .0006 |
| 4 | .5003 | .2676 | .3615 | .0487 |
| 4 | .3688 | .3517 | .1798 | .0811 |
| 4 | .5078 | .1954 | .3402 | .0451 |
| 4 | .5631 | .4299 | .2549 | .0875 |
| 4 | .4996 | .0132 | .2593 | .0516 |
| 4 | .3919 | .3140 | .2965 | .0845 |
| 4 | .3416 | .4391 | .2317 | .0363 |
| 4 | .6138 | .2871 | .3209 | .0169 |

**Table5** Sex, age, pure tone audiometry and speech discrimination score in patients with tinnitus or sensorineural hearing loss of the left ear.

| Group  （1=control，2=tinnitus，3=hearing loss，4=tinnitus+hearing loss） | Sex（F=female，M=male） | Age(years) | Pure tone audiometry(dBHL) | speech discrimination score |
| --- | --- | --- | --- | --- |
| 1 | M | 32 | 10 | 100 |
| 1 | M | 50 | 15 | 100 |
| 1 | M | 47 | 19 | 100 |
| 1 | M | 42 | 16 | 100 |
| 1 | M | 49 | 20 | 100 |
| 1 | M | 38 | 5 | 100 |
| 1 | M | 18 | 20 | 100 |
| 1 | M | 31 | 20 | 100 |
| 1 | M | 36 | 16 | 100 |
| 1 | M | 48 | 18 | 100 |
| 1 | F | 36 | 20 | 100 |
| 1 | F | 43 | 15 | 100 |
| 1 | F | 45 | 14 | 100 |
| 1 | F | 44 | 10 | 100 |
| 1 | F | 36 | 10 | 100 |
| 1 | F | 23 | 5 | 100 |
| 1 | F | 47 | 20 | 100 |
| 1 | F | 47 | 16 | 100 |
| 1 | F | 40 | 12 | 100 |
| 1 | F | 48 | 15 | 100 |
| 1 | F | 23 | 19 | 100 |
| 2 | M | 27 | 20 | 100 |
| 2 | M | 41 | 23 | 100 |
| 2 | M | 50 | 24 | 100 |
| 2 | M | 46 | 21 | 100 |
| 2 | M | 47 | 20 | 100 |
| 2 | F | 32 | 16 | 100 |
| 2 | F | 39 | 15 | 100 |
| 2 | F | 44 | 9 | 100 |
| 2 | F | 26 | 6 | 100 |
| 2 | F | 28 | 8 | 100 |
| 2 | F | 50 | 13 | 100 |
| 2 | F | 45 | 20 | 100 |
| 2 | F | 36 | 16 | 100 |
| 3 | M | 35 | 48 | 90 |
| 3 | M | 46 | 43 | 82 |
| 3 | M | 43 | 34 | 100 |
| 3 | M | 34 | 47 | 80 |
| 3 | M | 30 | 39 | 85 |
| 3 | M | 45 | 37 | 90 |
| 3 | F | 38 | 46 | 80 |
| 3 | F | 26 | 34 | 80 |
| 3 | F | 37 | 33 | 100 |
| 3 | F | 46 | 54 | 79 |
| 3 | F | 22 | 36 | 75 |
| 3 | F | 40 | 47 | 77 |
| 4 | M | 34 | 43 | 80 |
| 4 | M | 38 | 42 | 80 |
| 4 | M | 21 | 40 | 70 |
| 4 | M | 26 | 42 | 70 |
| 4 | M | 50 | 32 | 90 |
| 4 | M | 39 | 58 | 90 |
| 4 | F | 46 | 36 | 100 |
| 4 | F | 40 | 33 | 100 |
| 4 | F | 36 | 42 | 90 |
| 4 | F | 27 | 40 | 90 |
| 4 | F | 33 | 34 | 100 |
| 4 | F | 25 | 40 | 100 |
| 4 | F | 39 | 30 | 90 |
| 4 | F | 28 | 60 | 80 |
| 4 | F | 18 | 35 | 90 |

**Table6**  The MMN amplitude and latency in patients with tinnitus or sensorineural hearing loss of the left ear.

| Group  （1=control，2=tinnitus，3=hearing loss，4=tinnitus+hearing loss） | Amplitude(µV) | Latency(ms) |
| --- | --- | --- |
| 1 | -1.5 | 222.8 |
| 1 | -2.2 | 225.7 |
| 1 | -1.9 | 206.8 |
| 1 | -2.1 | 201.1 |
| 1 | -2.2 | 205.7 |
| 1 | -2.2 | 213.8 |
| 1 | -2 | 221.4 |
| 1 | -2.5 | 196.8 |
| 1 | -2.8 | 205.6 |
| 1 | -3.1 | 228.1 |
| 1 | -2.4 | 213.4 |
| 1 | -3.1 | 210 |
| 1 | -1.5 | 215.7 |
| 1 | -3.6 | 219.3 |
| 1 | -1.9 | 209.7 |
| 1 | -2.1 | 223.4 |
| 1 | -1.2 | 215.6 |
| 1 | -3.1 | 224.8 |
| 1 | -2.9 | 206.2 |
| 1 | -2.2 | 206.1 |
| 1 | -3.1 | 203.1 |
| 2 | -1.7 | 210.8 |
| 2 | -0.9 | 212.3 |
| 2 | -0.3 | 209.9 |
| 2 | -1 | 207.6 |
| 2 | -0.5 | 207.3 |
| 2 | -1.3 | 210.2 |
| 2 | -1.2 | 230.5 |
| 2 | -2.2 | 212.1 |
| 2 | -1.2 | 227.3 |
| 2 | -1.6 | 212.5 |
| 2 | -0.8 | 209.9 |
| 2 | -0.7 | 225 |
| 2 | -0.2 | 210.1 |
| 3 | -0.4 | 281.6 |
| 3 | -0.7 | 278.2 |
| 3 | -0.8 | 282.3 |
| 3 | -1 | 283.3 |
| 3 | -0.4 | 282.2 |
| 3 | -0.8 | 284.6 |
| 3 | -0.8 | 280.9 |
| 3 | -0.5 | 277.2 |
| 3 | -1.1 | 288.8 |
| 3 | -0.9 | 272 |
| 3 | -0.9 | 281.8 |
| 3 | -0.5 | 276.9 |
| 4 | -0.3 | 264.7 |
| 4 | -1.1 | 269 |
| 4 | -1 | 274.9 |
| 4 | -1.1 | 284.2 |
| 4 | -1.4 | 279 |
| 4 | -0.5 | 274.1 |
| 4 | -0.7 | 279 |
| 4 | -1 | 282.1 |
| 4 | -0.7 | 283.9 |
| 4 | -1.3 | 279.8 |
| 4 | -0.8 | 275.8 |
| 4 | -1.3 | 271.2 |
| 4 | -1.4 | 275.4 |
| 4 | -1.4 | 277.6 |
| 4 | -0.5 | 271.8 |

**Table 7** The clustering coefficient, average path length and functional connectivity of AC, PHG and IFG in the left hemisphere in patients with tinnitus or sensorineural hearing loss of the left ear.

| Group  （1=control，2=tinnitus，3=hearing loss，4=tinnitus+hearing loss） | C | Lw | AC→PHG | AC→IFG | PHG→IFG | IFG→AC |
| --- | --- | --- | --- | --- | --- | --- |
| 1 | .0530 | 4.7294 | .4196 | .5560 | .4186 | .0617 |
| 1 | .0504 | 3.3348 | .3466 | .4014 | .4560 | .0421 |
| 1 | .0713 | 4.8687 | .4710 | .3190 | .5339 | .0820 |
| 1 | .0566 | 4.0226 | .5280 | .5320 | .4509 | .0855 |
| 1 | .0532 | 3.7283 | .4708 | .4192 | .4969 | .0855 |
| 1 | .0451 | 3.7807 | .5478 | .3876 | .5228 | .0717 |
| 1 | .0623 | 3.9456 | .5302 | .3154 | .4740 | .0480 |
| 1 | .0803 | 3.8453 | .4460 | .3330 | .6507 | .0062 |
| 1 | .0750 | 4.8775 | .5795 | .4130 | .5800 | .0899 |
| 1 | .0850 | 4.2549 | .5350 | .5057 | .7132 | .0582 |
| 1 | .0590 | 3.4236 | .5280 | .5790 | .5886 | .0552 |
| 1 | .0530 | 3.6165 | .3224 | .3024 | .7230 | .0439 |
| 1 | .0570 | 4.8392 | .5640 | .4779 | .4046 | .0894 |
| 1 | .0643 | 4.8232 | .4122 | .6673 | .4232 | .0915 |
| 1 | .0639 | 3.3548 | .5810 | .5189 | .4479 | .0260 |
| 1 | .0619 | 3.6618 | .4992 | .4832 | .5057 | .0727 |
| 1 | .0680 | 4.0916 | .3391 | .4995 | .4134 | .0479 |
| 1 | .0400 | 3.5000 | .6050 | .3802 | .4926 | .0808 |
| 1 | .0594 | 3.0787 | .4242 | .3474 | .4815 | .0332 |
| 1 | .0637 | 3.8802 | .6175 | .3278 | .8241 | .0903 |
| 1 | .0560 | 4.4798 | .4360 | .4179 | .6715 | .0065 |
| 2 | .0240 | 6.7546 | .0977 | .0856 | .0984 | .1462 |
| 2 | .0121 | 5.1884 | .0974 | .0900 | .0965 | .1509 |
| 2 | .0260 | 6.2426 | .0966 | .0863 | .0839 | .1385 |
| 2 | .0310 | 5.6291 | .0969 | .0838 | .0976 | .1350 |
| 2 | .0238 | 5.8234 | .0812 | .0793 | .0949 | .2067 |
| 2 | .0270 | 6.4634 | .0967 | .0869 | .0965 | .1064 |
| 2 | .0365 | 6.5130 | .0974 | .0876 | .0848 | .1447 |
| 2 | .0156 | 5.6918 | .0857 | .0854 | .0974 | .1957 |
| 2 | .0360 | 6.6078 | .0860 | .0851 | .0960 | .1165 |
| 2 | .0222 | 5.8699 | .0625 | .0709 | .0737 | .1329 |
| 2 | .0128 | 5.7904 | .0931 | .0683 | .0820 | .1220 |
| 2 | .0114 | 5.2504 | .0675 | .0504 | .0903 | .1410 |
| 2 | .0296 | 5.9622 | .0774 | .0662 | .0844 | .1353 |
| 3 | .0088 | 6.1705 | .0412 | .0150 | .0088 | .5083 |
| 3 | .0105 | 6.6355 | .0434 | .0257 | .0559 | .4953 |
| 3 | .0104 | 6.5765 | .0358 | .0279 | .0295 | .4021 |
| 3 | .0114 | 5.8142 | .0281 | .0262 | .0315 | .4374 |
| 3 | .0078 | 6.7221 | .0407 | .0174 | .0080 | .4487 |
| 3 | .0081 | 6.3900 | .0454 | .0319 | .0049 | .5903 |
| 3 | .0117 | 6.9366 | .0353 | .0231 | .0270 | .3811 |
| 3 | .0116 | 7.1910 | .0294 | .0320 | .0223 | .8311 |
| 3 | .0112 | 6.9755 | .0415 | .0314 | .0375 | .4274 |
| 3 | .0130 | 6.2829 | .0522 | .0165 | .0094 | .7362 |
| 3 | .0077 | 7.5018 | .0494 | .0205 | .0165 | .3918 |
| 3 | .0081 | 6.8233 | .0504 | .0121 | .0148 | .4124 |
| 4 | .0069 | 6.4693 | .0250 | .0194 | .0211 | .5995 |
| 4 | .0071 | 6.8290 | .0312 | .0111 | .0100 | .5637 |
| 4 | .0063 | 7.7194 | .0350 | .0299 | .0162 | .6959 |
| 4 | .0093 | 7.8342 | .0409 | .0171 | .0125 | .4256 |
| 4 | .0085 | 6.6630 | .0258 | .0286 | .0311 | .6997 |
| 4 | .0077 | 6.3856 | .0293 | .0261 | .0177 | .7500 |
| 4 | .0075 | 7.2426 | .0240 | .0351 | .0460 | .5930 |
| 4 | .0085 | 6.7076 | .0396 | .0208 | .0370 | .5221 |
| 4 | .0083 | 6.6675 | .0490 | .0171 | .0125 | .6474 |
| 4 | .0057 | 6.3210 | .0412 | .0110 | .0161 | .7410 |
| 4 | .0061 | 6.6640 | .0340 | .0249 | .0317 | .4494 |
| 4 | .0069 | 6.6318 | .0302 | .0181 | .0326 | .4648 |
| 4 | .0094 | 7.9425 | .0350 | .0217 | .0350 | .4030 |
| 4 | .0081 | 6.2059 | .0363 | .0233 | .0306 | .4313 |
| 4 | .0054 | 6.7705 | .0335 | .0159 | .0295 | .5200 |

**Table 8** The functional connectivity from the left hemisphere to the right hemisphere in patients with tinnitus or sensorineural hearing loss of the left ear.

| Group  （1=control，2=tinnitus，3=hearing loss，4=tinnitus+hearing loss） | AC→PHG | AC→IFG | PHG→IFG | IFG→AC |
| --- | --- | --- | --- | --- |
| 1 | .2421 | .6355 | .6949 | .0605 |
| 1 | .5625 | .6389 | .4774 | .0956 |
| 1 | .4839 | .4242 | .3953 | .0666 |
| 1 | .3539 | .2865 | .6221 | .0523 |
| 1 | .3983 | .6742 | .5562 | .0187 |
| 1 | .4926 | .4101 | .5119 | .0800 |
| 1 | .3703 | .4542 | .5367 | .0716 |
| 1 | .4539 | .6006 | .4999 | .0547 |
| 1 | .3225 | .4978 | .6437 | .0691 |
| 1 | .3548 | .4327 | .6606 | .0705 |
| 1 | .5106 | .4084 | .2511 | .0873 |
| 1 | .4765 | .5810 | .6451 | .0434 |
| 1 | .3530 | .4695 | .3796 | .0474 |
| 1 | .5022 | .4001 | .4419 | .0011 |
| 1 | .3578 | .4590 | .5823 | .0794 |
| 1 | .5506 | .3097 | .5046 | .0347 |
| 1 | .5777 | .7084 | .5356 | .0514 |
| 1 | .5138 | .4542 | .4303 | .0681 |
| 1 | .4552 | .6141 | .6696 | .0517 |
| 1 | .4364 | .5278 | .5317 | .0706 |
| 1 | .3982 | .3287 | .4016 | .0029 |
| 2 | .5841 | .3618 | .3912 | .0507 |
| 2 | .4720 | .3129 | .4687 | .0494 |
| 2 | .3756 | .3692 | .5133 | .0395 |
| 2 | .4481 | .5329 | .6275 | .1070 |
| 2 | .5182 | .3703 | .4230 | .0763 |
| 2 | .2933 | .4954 | .4965 | .0890 |
| 2 | .4615 | .5590 | .4409 | .0871 |
| 2 | .4743 | .4460 | .6101 | .0598 |
| 2 | .4145 | .5305 | .4986 | .0611 |
| 2 | .2763 | .6322 | .7158 | .0817 |
| 2 | .5416 | .4975 | .6053 | .0299 |
| 2 | .4123 | .4581 | .7279 | .0554 |
| 2 | .4263 | .2739 | .7487 | .0744 |
| 3 | .4282 | .4671 | .4646 | .0060 |
| 3 | .4462 | .3693 | .4076 | .0756 |
| 3 | .5348 | .4751 | .3959 | .0559 |
| 3 | .5637 | .5063 | .3721 | .0589 |
| 3 | .4697 | .5752 | .5673 | .0305 |
| 3 | .3536 | .4438 | .5864 | .0584 |
| 3 | .3946 | .4812 | .5000 | .1042 |
| 3 | .4599 | .5280 | .5014 | .0594 |
| 3 | .6910 | .4011 | .6519 | .0704 |
| 3 | .6023 | .4371 | .2241 | .0866 |
| 3 | .3522 | .4417 | .5011 | .0681 |
| 3 | .3758 | .4219 | .6153 | .1081 |
| 4 | .4989 | .5525 | .4953 | .0668 |
| 4 | .3940 | .4483 | .5353 | .0625 |
| 4 | .3600 | .4494 | .5943 | .0466 |
| 4 | .5404 | .5041 | .5240 | .0278 |
| 4 | .3914 | .4957 | .4132 | .0887 |
| 4 | .2965 | .3740 | .4777 | .0598 |
| 4 | .4120 | .5356 | .5217 | .0440 |
| 4 | .4606 | .5630 | .6258 | .0373 |
| 4 | .4934 | .2160 | .5774 | .0663 |
| 4 | .4436 | .5928 | .6360 | .0501 |
| 4 | .4876 | .5267 | .4322 | .0657 |
| 4 | .4397 | .8007 | .6524 | .0391 |
| 4 | .3677 | .6596 | .6845 | .0508 |
| 4 | .3040 | .2587 | .7126 | .0508 |
| 4 | .3242 | .3644 | .6895 | .0644 |
