# Supplementary material for: Sensorineural Hearing Loss Affects Functional Connectivity of the Auditory Cortex, Parahippocampal Gyrus and Inferior Prefrontal Gyrus in Tinnitus Patients
Source: Front Neurosci. 2022 Apr 1;16:816712. doi: 10.3389/fnins.2022.816712 (PMC9011051; doi:10.3389/fnins.2022.816712)
Supplement: Supplementary file 2 [file Table_2.docx]

**The matlab code of the synchronization likelihood, clustering coefficient, average path length and Granger causality**

**The caculation of the synchronization likelihood**

clear

clc

w1=20.8;w2=30;Pref=0.05;

load('.mat')

STA=;

[n,m]=size(STA);

ljjl_min=zeros(n,n);

ljjl_max=zeros(n,n);

for k=1:n

jsjz=1./zeros(11,1);

for i=1:m

for j=1:m

if(abs(j-i)>w1&&abs(j-i)<w2&&abs(STA(k,i)-STA(k,j))<jsjz(11))

jsjz(11)=abs(STA(k,i)-STA(k,j));

for l=1:10

if(jsjz(l)>jsjz(l+1))

a=jsjz(l);

jsjz(l)=jsjz(l+1);

jsjz(l+1)=a;

end

end

end

end

ljjl_max(k,i)=jsjz(10);

ljjl_min(k,i)=jsjz(11);

end

end

M=n;

H=zeros(m,m);

for i=1:m

for j=1:m

for k=1:M

if(ljjl_max(k,i)-abs(STA(k,i)-STA(k,j))>0)

H(i,j)=H(i,j)+1;

end

end

end

end

S_kij=zeros(M,m,m);

for k=1:M

for i=1:m

for j=1:m

if(abs(STA(k,i)-STA(k,j))<ljjl_max(k,i))

S_kij(k,i,j)=(H(i,j)-1)/(M-1);

end

end

end

end

S_kij_sum=zeros(M,m);

S_ki=zeros(M,m);

for k=1:M

for i=1:m

for j=1:m

if(abs(j-i)>w1&&abs(j-i)<w2)

S_kij_sum(k,i)=S_kij_sum(k,i)+S_kij(k,i,j);

end

end

S_ki(k,i)=1/(2*(w2-w1))*(S_kij_sum(k,i));

end

end

s_tblj=zeros(n,n);

S=zeros(n,n);

for a=1:n

for b=1:n

for i=1:m

for j=1:m

if((ljjl_max(a,i)-abs(STA(a,i)-STA(a,j)))>0&&(ljjl_max(b,i)-abs(STA(b,i)-STA(b,j)))>0&&abs(j-i)>w1&&abs(j-i)<w2)

s_tblj(a,b)=s_tblj(a,b)+1;

end

end

end

end

end

for a=1:n

for b=1:n

S(a,b)=S(a,b)+1/2/Pref/(w2-w1)*s_tblj(a,b);

end

end

[n,m]=size(S);

Ks=zeros(1,n);

Cs=zeros(n,n);

for k=1:n

for j=1:n

cs=cov(S(k,:),S(j,:));

if(sqrt(cs(1,1))==0||sqrt(cs(2,2))==0)

Cs(k,j)=0;

else

Cs(k,j)=cs(1,2)/sqrt(cs(1,1))/sqrt(cs(2,2));

end

if(Cs(k,j)>=0.90&&k~=j)

Ks(k)=Ks(k)+1;

end

end

end

**The caculation of the clustering coefficient**

Es=zeros(n);

KZs=zeros(n,n);

Cis=zeros(n,1);

for k=1:n

ps=1;

for j=1:n

if(Cs(k,j)>=0.95&&k~=j)

KZs(k,ps)=j;

ps=ps+1;

end

end

if(ps>1)

for j=1:ps-2

for m1=j+1:ps-1

if(Cs(KZs(k,j),KZs(k,m1))>=0.95)

Es(k)=Es(k)+1;

end

end

end

end

if(Ks(k)==0||(Ks(k)==1))

Cis(k)=0;

else

Cis(k)=2*Es(k)/((Ks(k))*(Ks(k)-1));

end

end

Cjs=0;

for k=1:n

Cjs=Cjs+Cis(k)/n;

end

**The caculation of the average path length**

sum_Lijs=0;

for i1=1:n

for j1=1:n

if(i1~=j1)

sum_Lijs=sum_Lijs+abs(Cs(i1,j1));

end

end

end

if(sum_Lijs==0)

Lws=0;

else

Lws=n*(n-1)/sum_Lijs;

end

**The matlab code of the Granger causality**

clear

clc

load('.mat')

Y=;

[n,m]=size(Y);

AIC=zeros(1,m);

m1=m;

BL=1;

if n<m

m1=n;

end

for i=1:m1

Y1=zeros(i,i);

for k=1:i

for l=1:i

Y1(k,l)=Y(k,l);

end

end

AIC(i)=m*log(det(cov(Y1)))+2*i*BL^2;

end

zxz=abs(AIC(1));

js=1;

for i=2:m1

if(zxz>abs(AIC(i)))

js=i;

zxz=abs(AIC(i));

end

end

js=js+1;

Y2=zeros(m-js,1);

C=zeros(n,js);%

for k=1:n

M=zeros(m-js,js);

for i=1+js:m

for j=1:js

M(i-js,j)=Y(k,i-j);

end

end

for j=js+1:m

Y2(j-js)=Y(k,j);

end

c=inv(M'*M)*M'*Y2;

for z=1:js

C(k,z)=c(z);

end

end

E=zeros(n,m-js);

for i=1:n

for j=js+1:m

sum=0;

for k=1:js

sum=sum+C(i,k)*Y(i,j-k);

end

E(i,j-js)=abs(Y(i,j)-sum);

end

end

X=zeros(m-js,1);

Y3=zeros(m-js,1);

X_x=zeros(1,js*2);

Y_x=zeros(1,js*2);

E2=zeros(1,m-js);

E3=zeros(1,m-js);

a2=zeros(n,n,js);

b2=zeros(n,n,js);

c2=zeros(n,n,js);

d2=zeros(n,n,js);

P_X_Y=zeros(n,n);

P_Y_X=zeros(n,n);

r=zeros(n,n);

for i=1:n

for j=1:n

if(i==j)

continue;

end

M2=zeros(m-js,js+js);

for k=1:m-js

for l=k:k+js-1

M2(k,k+js-l)=Y(i,l);

M2(k,k+js*2-l)=Y(j,l);

end

end

for k=js+1:m

X(k-js)=Y(i,k);

end

for k=js+1:m

Y3(k-js)=Y(j,k);

end

X_x=inv(M2'*M2)*M2'*X;

Y_x=inv(M2'*M2)*M2'*Y3;

for k=js+1:m

sum2=0;

sum3=0;

for l=1:js

sum2=sum2+X_x(l)*Y(i,k-l)+X_x(l+js)*Y(j,k-l);

sum3=sum3+Y_x(l)*Y(i,k-l)+Y_x(l+js)*Y(j,k-l);

a2(i,j,l)=X_x(l);

b2(i,j,l)=X_x(l+js);

c2(i,j,l)=Y_x(l);

d2(i,j,l)=Y_x(l+js);

end

E2(k-js)=abs(Y(i,k)-sum2);

E3(k-js)=abs(Y(j,k)-sum3);

end

if(log(var(E(j,:))/var(E3))>0)

P_X_Y(i,j)=log(var(E(j,:))/var(E3(1,:)));

end

if(log(var(E(i,:))/var(E2))>0)

P_Y_X(i,j)=log(var(E(i,:))/var(E2(1,:)));

end

for k=1+js:m

E2t(i,j,k-js)=E2(k-js);

T2t(i,j,k-js)=E3(k-js);

end

end

end

**cca_detrend (removing deterministic linear trends)**

nobs = size(X,2);

nvar = size(X,1);

if(nvar>nobs) error('error in cca_detrend: nvar>nobs, check input matrix'); end

X = X';

Y = detrend(X);

Y = Y';

**cca_dif (removing unit roots)**

[nvar nobs] = size(M);

if(nobs < nvar)

error('error in cca_diff: nobs < nvar, check data matrix');

end

M2 = diff(M');

M2 = M2';

**cca_rm_ensemblemean**

[~,Nl]=size(X2);

Nr=5;

Nl=Nl/Nr;

FLAG=1;

[X3,M,E] = cca_rm_ensemblemean(X2,Nr,Nl,FLAG);

[nvar nobs] = size(X);

if(nobs < nvar)

error('error in cca_rm_ensemblemean: nobs < nvar, check data matrix');

end

if nargin < 3,

error('insufficient inputs to cca_rm_ensemblemean');

end

if nargin == 3,

FLAG = 0;

E = [];

end

if nobs ~= Nr*Nl,

error('cca_rm_ensemblemean: inputs do not match data matrix size');

end

% reshape matrix

XX = zeros(Nl,nvar,Nr);

for ii=1:nvar,

inx = 1;

for jj=1:Nr,

XX(:,ii,jj) = X(ii,inx:inx+Nl-1);

inx=inx+Nl;

end

end

% subtract ensemble mean

M = zeros(nvar,Nl);

if FLAG==1,

E =zeros(nvar,Nl);

end

for ii = 1:nvar,

temp = XX(:,ii,:);

m = mean(temp,3);

if FLAG==1, e = std(temp,0,3); end

for jj = 1:Nr,

XX(:,ii,jj) = XX(:,ii,jj)-m;

if FLAG==1,

XX(:,ii,jj) = XX(:,ii,jj)./e;

end

end

M(ii,:) = m;

if FLAG==1,

E(ii,:) = e;

end

end

% inverse reshape matrix

for ii=1:nvar,

inx = 1;

for jj=1:Nr,

X(ii,inx:inx+Nl-1) = XX(:,ii,jj);

inx=inx+Nl;

end

end

clear XX;

X2 = X;

**Calculate VAR model consistency Granger causality**

X = Y;

E = Y;

lags = 4;

for i = 1:lags

E(:,size(E,2))=[];

end

cons = consistency(X,E);

**Code of consistency**

function cons = consistency(X,E)

[n,m,N] = size(X);

p = m-size(E,2);

assert(m >= n,'too few observations');

assert(size(E,1) == n && size(E,3) == N,'residuals don''t match data');

assert(p > 0,'bad number of lags');

X = demean(X);

if N > 1 % multi-trial

X = X(:,p+1:m,:);

X = X(:,:);

E = E(:,:);

s = N*(m-p);

else

X = X(:,p+1:m);

s = m-p;

end

Y = X - E;

Rr = (X*X')/(s-1);

Rs = (Y*Y')/(s-1);

cons = 1 - norm(Rs-Rr)/norm(Rr);

**Code of demean for consistency**

function Y = demean(X,normalise)

if nargin < 2 || isempty(normalise), normalise = false; end

[n,m,N] = size(X);

U = ones(1,N*m);

Y = X(:,:);

Y = Y-mean(Y,2)*U;

if normalise

Y = Y./(std(Y,[],2)*U);

end

Y = reshape(Y,n,m,N);
